# Regulation of Galactolipid Biosynthesis by Overexpression of the Rice *MGD* Gene Contributes to Enhanced Aluminum Tolerance in Tobacco

**DOI:** 10.3389/fpls.2016.00337

**Published:** 2016-03-30

**Authors:** Meijuan Zhang, Xiping Deng, Lina Yin, Lingyun Qi, Xinyue Wang, Shiwen Wang, Hongbing Li

**Affiliations:** ^1^State Key Laboratory of Soil Erosion and Dryland Farming on the Loess Plateau, Institute of Soil and Water Conservation, Northwest A&F UniversityYangling, China; ^2^College of Life Sciences, Northwest A&F UniversityYangling, China; ^3^Institute of Soil and Water Conservation, Chinese Academy of Sciences and Ministry of Water ResourcesYangling, China; ^4^College of Natural Resources and Environment, Northwest A&F UniversityYangling, China

**Keywords:** aluminum, monogalactosyldiacylglycerol, galactolipid, phospholipid, membrane integrity

## Abstract

Membrane lipid alterations affect Al tolerance in plants, but little is known about the regulation of membrane lipid metabolism in response to Al stress. Transgenic tobacco (*Nicotiana tabacum*) overexpressing rice monogalactosyldiacylglycerol (MGDG) synthase (*OsMGD*) gene and wild-type tobacco plants were exposed to AlCl_3_, and the impact of Al toxicity on root growth, Al accumulation, plasma membrane integrity, lipid peroxidation and membrane lipid composition were investigated. Compared with the wild type, the transgenic plants exhibited rapid regrowth of roots after removal of Al and less damage to membrane integrity and lipid peroxidation under Al stress, meanwhile, the Al accumulation showed no difference between wild-type and transgenic plants. Lipid analysis showed that Al treatment dramatically decreased the content of MGDG and the ratio of MGDG to digalactosyldiacylglycerol (DGDG) in wild-type plants, while it was unchanged in transgenic plants. The stable of MGDG level and the ratio of MGDG/DGDG contribute to maintain the membrane stability and permeability. Moreover, Al caused a significant increase in phospholipids in wild-type plants, resulting in a high proportion of phospholipids and low proportion of galactolipids, but these proportions were unaffected in transgenic plants. The high proportion of phospholipids could contribute to a higher rate of Al^3+^ binding in the membrane and thereby leads to more membrane perturbation and damage. These results show that the regulation of galactolipid biosynthesis could play an important role in maintaining membrane structure and function under Al stress.

## Introduction

Aluminum (Al) is the most abundant metal in the earth's crust and is a major factor limiting plant production in acid soils, which cover about 50% of the world's potentially arable land surface (Kochian et al., 2004; Liu et al., 2014). Various studies have been focused on plant response to Al stress, including the molecular, genetic, and physiological bases for Al tolerance (Poschenrieder et al., 2008; Kochian et al., 2015). Under low pH condition, the toxic Al cations (particularly Al^3+^) can be easily released from Al-containing compounds and thus inhibits root elongation rapidly by suppressing cell expansion and division, resulting in a damaged root system and indirectly limited water and nutrient uptake (Silva, 2012). Meanwhile, toxic Al cations can interact with a number of extracellular and intracellular substances, causing various impacts on plant growth, including alteration of cell wall properties, generation of reactive oxygen species (ROS) and affection of the apoplastic processes (Panda and Matsumoto, 2007; Horst et al., 2010).

Cell membranes are vital because they separate the cell from its surrounding environment and enable cellular activities to proceed without external interference. However, they are easily damaged by various environmental stresses, including Al stress. It has been reported that cell membranes are the primary target of Al toxicity, since Al disturbs membrane stability and integrity very quickly and eventually inhibits plant growth (Krtková et al., 2012; Too et al., 2014). Lipids are the predominant constituent of cell membranes, and changes in membrane lipid composition have frequently been found under various environmental stress conditions, such as cold, drought and salinity; these changes are thought to contribute to the restoration and maintenance of membrane stability and integrity, and thus to increase plant stress tolerance (Campos et al., 2003; Gigon et al., 2004; Bybordi, 2011). Previous studies showed that the content of phospholipids decreased significantly under drought and salt stresses in wheat (Mansour et al., 2002; El Kaoua et al., 2006). Lauriano et al. (2000) found that changes in lipid composition were different between sensitive and tolerant peanut cultivars: the contents of galactolipids, including monogalactosyldiacylglycerol (MGDG) and digalactosyldiacylglycerol (DGDG), and phospholipids, including phosphatidylcholine (PC), phosphatidylglycerol (PG), and phosphatidylinositol (PI), were significantly decreased in the sensitive cultivars, while their contents were decreased less or unchanged in the tolerant one under drought stress, and that the loss of membrane integrity was less severe in tolerant cultivar. Recently, our group has found that increased the biosynthesis of MGDG contributes to maintain the membrane structure and function of chloroplast, and thus leads to enhanced salt tolerance (Wang et al., 2014).

Although the impact of Al toxicity on lipid composition of cell membranes has only rarely been reported to date, the few available studies have suggested that membrane lipids are involved in the plant response to Al (Zhang et al., 1996; Huynh et al., 2012). It was reported that the levels of several phospholipids, including PC, PI, phosphatidylethanolamine (PE), and phosphatidylserine (PS), were increased in roots of Arabidopsis seedlings when exposed to Al stress (Zhao et al., 2011). In maize, the contents of MGDG and PC were increased in roots, while in shoots, the contents of MGDG, DGDG, and PC were significantly decreased under Al stress (Chaffai et al., 2005). Furthermore, it was also found that the changes in membrane lipid composition were different in Al-sensitive and Al-tolerant cultivars, suggesting a close relationship between membrane lipid composition and plant Al tolerance capability (Zhang et al., 1996, 1997; Huynh et al., 2012). For example, phospholipids (particularly PC) and MGDG decreased significantly after Al treatment in the roots of Al-sensitive rice cultivars, whereas the amount of lipid classes remained unchanged in the tolerant ones (Huynh et al., 2012). Taken together, these findings indicate that Al can cause changes in membrane lipid composition and that these alterations are involved in plant response to Al stress.

Galactolipids are the important membrane lipid constituents in plants. Although they are not commonly believed to be the major components of non-plastidic membranes, changes in these membrane components are often observed under stress conditions, and such changes have been shown to play an important role in the acquisition of stress tolerance. Several studies have demonstrated that the accumulation of galactolipids could allow them to replace phospholipids under phosphate (Pi) starvation condition and that this process plays a key role in the plant's ability to adapt to Pi starvation condition (Andersson et al., 2003; Jouhet et al., 2004; Shimojima et al., 2013; Maejima et al., 2014). Our previous study showed that galactolipids are essential for phosphorus dificiency tolerance in plants (Shi et al., 2013). It is known that Al stress often accompanies with Pi starvation since Al ion can easily bind with the negative sites of the phosphate groups of phospholipids (Clarkson, 1966; Deleers et al., 1986; MacKinnon et al., 2004; Maejima et al., 2014), thus, the increase of galactolipids biosynthesis may also contribute to Al tolerance. Moreover, it was found that the tolerant wheat cultivar has higher MGDG content than the sensitive one when exposed to Al stress (Zhang et al., 1996, 1997), indicating that these galactolipids are involved and may play an important role in plant Al tolerance. However, the underlying mechanisms by which galactolipids contribute to regulating plant Al tolerance are still unclear.

To gain insight into the role of galactolipids in plant Al tolerance, transgenic tobacco plants overexpressing rice monogalactosyldiacylglycerol synthase (*OsMGD*) gene, which encodes the key enzyme for galactolipid biosynthesis, and wild-type tobacco plants were used. Root growth, membrane integrity, membrane lipid contents, and fatty acid compositions were investigated in transgenic and wild-type plants, and the possible relationships between changes in membrane lipid compositions and Al tolerance were discussed.

## Materials and methods

### Plant materials and growth conditions

Transgenic tobacco plants overexpressing the rice *MGD* gene (*OsMGD*, AB112060) were generated by Wang et al. (2014). The expression of *OsMGD* in these transgenic plants has been verified by genomic PCR and western-blot analysis. Moreover, the MGD activity in *OsMGD* transgenic plants was to be higher than that in the wild-type SR1 (Wang et al., 2014).

Seeds of tobacco (*Nicotiana tabacum*: wild-type SR1 and transgenic lines MGD3 and MGD5) were surface-sterilized in 1% (w/v) sodium hypochlorite for 20 min, then grown on MS (Murashige and Skoog, 1962) agar plates (pH 5.7) containing 3% sucrose and 0 (for wild-type SR1) or 50 mg L^−1^ hygromycin (for transgenic lines) for 4 weeks. The plants were then transferred to aerated one-sixth strength Hoagland solution (HS) (pH 5.7) and maintained in a growth chamber kept at 25°C with a 14 h photoperiod at 200 μmol photons m^−2^ s^−1^ for another 4 weeks.

### Al treatment

Before Al treatment, the uniformly grown plants at the five- to six-leaf stage were selected and precultivated for 24 h at pH 4.2 in one-sixth strength HS. Thereafter, the plants were exposed to 0 μM (control) or 500 μM AlCl_3_ (Al treatment) for 24 h in the same solution at pH 4.2 to limit Al precipitation. After Al treatment, one set of the seedlings were retransplanted into well-aerated one-sixth strength HS (pH 5.7) without AlCl_3_ and kept for 3 days for recovery, and the root fresh weights were measured at each time point. The histochemical staining of roots was carried out immediately after sampling. For determination of the levels of Al accumulation, lipid peroxidation, electrolyte leakage and lipid compositions, roots and leaves were sampled in control and stressed plants.

### Al accumulation in root tips

For analysis of Al accumulation in the root tips, the last 0–20 mm of root tips from the roots with similar length were was washed three times with distilled water and dried, and the Al content was measured using an inductively coupled plasma atomic emission spectrometer (ICP-AES, Ciros CCD, Rigaku, Japan) according to the method of Yin et al. (2010b). The experiment was repeated three times and each treatment included three replications.

### Visualization of plasma membrane integrity

After Al treatment, root tips (0–20 mm, from the roots with similar length) exposed to 0 μM or 500 μM AlCl_3_ were excised and stained immediately with 0.025% (w/v) Evans blue (Sigma) solution (in 100 μM CaCl_2_ solution, pH 5.6) for 10 min (Yamamoto et al., 2001). Stained roots were washed three times with 100 μM CaCl_2_ (pH 5.6) until the dye no longer eluted from the roots, and then observed under a light stereomicroscope (Olympus BX-51, Japan). A total of 10–15 individual roots from five individual seedlings were examined, and the experiment was repeated three times.

Plasma membrane integrity was quantified in terms of electrolyte leakage (EL) by measuring changes in electrical conductivity (Singh et al., 2007). Root tips (0.1 g, 0–20 mm) were incubated in distilled water at 25°C for 2 h in tubes, and the initial electrical conductivity (E1) of the medium was measured. The tubes containing the root material were then boiled for 30 min to release all the electrolytes, then cooled to 25°C, and the final electrical conductivity (E2) was measured. The EL was calculated as EL = (E1/E2) × 100%. The experiment was repeated three times and each treatment included three replications.

### Determination of lipid peroxidation

Lipid peroxidation was estimated in the root tips (0–20 mm, from the roots with similar length) by measuring the malondialdehyde (MDA) content, as described in the TBARS methods (Heath and Packer, 1968). The roots were frozen in liquid nitrogen and ground with a mortar and pestle in 5 mL precooled 0.1% (w/v) trichloroacetic acid (TCA) solution. The homogenate was centrifuged at 12,000 × g for 15 min, with the addition of 1 mL 0.6% (w/v) TBA in 20% TCA to one volume of the supernatant. The mixture was incubated in boiling water for 30 min, and the reaction was stopped by placing the reaction tubes in an ice bath. Thereafter, the samples were centrifuged at 10,000 × g for 5 min, and the absorbance of the supernatant was measured at 532 nm and corrected by subtracting the non-specific absorbance at 600 nm. The MDA content was calculated using 155 mM^−1^cm^−1^ as an extinction coefficient. The experiment was repeated three times and each treatment included three replications.

### Lipid analysis

Lipids were extracted according to the method initially described by Bligh and Dyer (1959) and modified by Wewer et al. (2013). Each frozen root tips (0.5 g, 0–30 mm root tips from the roots with similar length) was homogenized in liquid nitrogen with 5 mL of chloroform/methanol/formic acid (1:1:0.1, v/v/v); the homogenate was collected and shaken vigorously. Subsequently, 2.5 mL of 1 M KCl/0.2 M H_3_PO_4_ was added and the mixture was vortexed briefly. The homogenized samples were centrifuged at 4000 × g for 3 min, and the lower chloroform layer was transferred to a new vial. Extraction was repeated by adding 5 mL of chloroform/methanol (2:1, v/v) to the residue, shaking and centrifuging the mixture, and gathering the chloroform phase. The combined chloroform phases were evaporated with a stream of nitrogen, and 500 μL of chloroform were added. The samples were then stored at −20°C until analysis.

Lipids were separated by Thin Layer Chromatography (TLC) on silica gel plates (G60; Merck, Germany) according to Wang and Benning (2011). After stained with iodine (see Supplementary Figure 1 for the separation of lipid by TLC), the identified lipid bands were scraped off with a razor blade and placed into tubes. Then, the lipid was methylated with HCl in methanol and converted into fatty acyl methylester (FAME), and the resulting FAMEs were quantified by gas chromatography (GC-2010; Shimadzu, Japan) with flame ionization detector (FID) according to Wang and Benning (2011) and Wewer et al. (2013). Pentadecanoic acid (15:0) was used as an internal standard.

### Statistical analyses

Statistical analysis was performed with SPSS-16 statistical software. Means were compared by analysis of variance (ANOVA), and differences were protected with the least significant difference (LSD) test. Three independent experiments were conducted for the measurement of root fresh weight, hematoxylin staining, Al content, Evans blue staining, electrolyte leakage and MDA content. Lipid contents were measured twice.

## Results

### Effect of Al treatment on root growth

**Figure 1:**
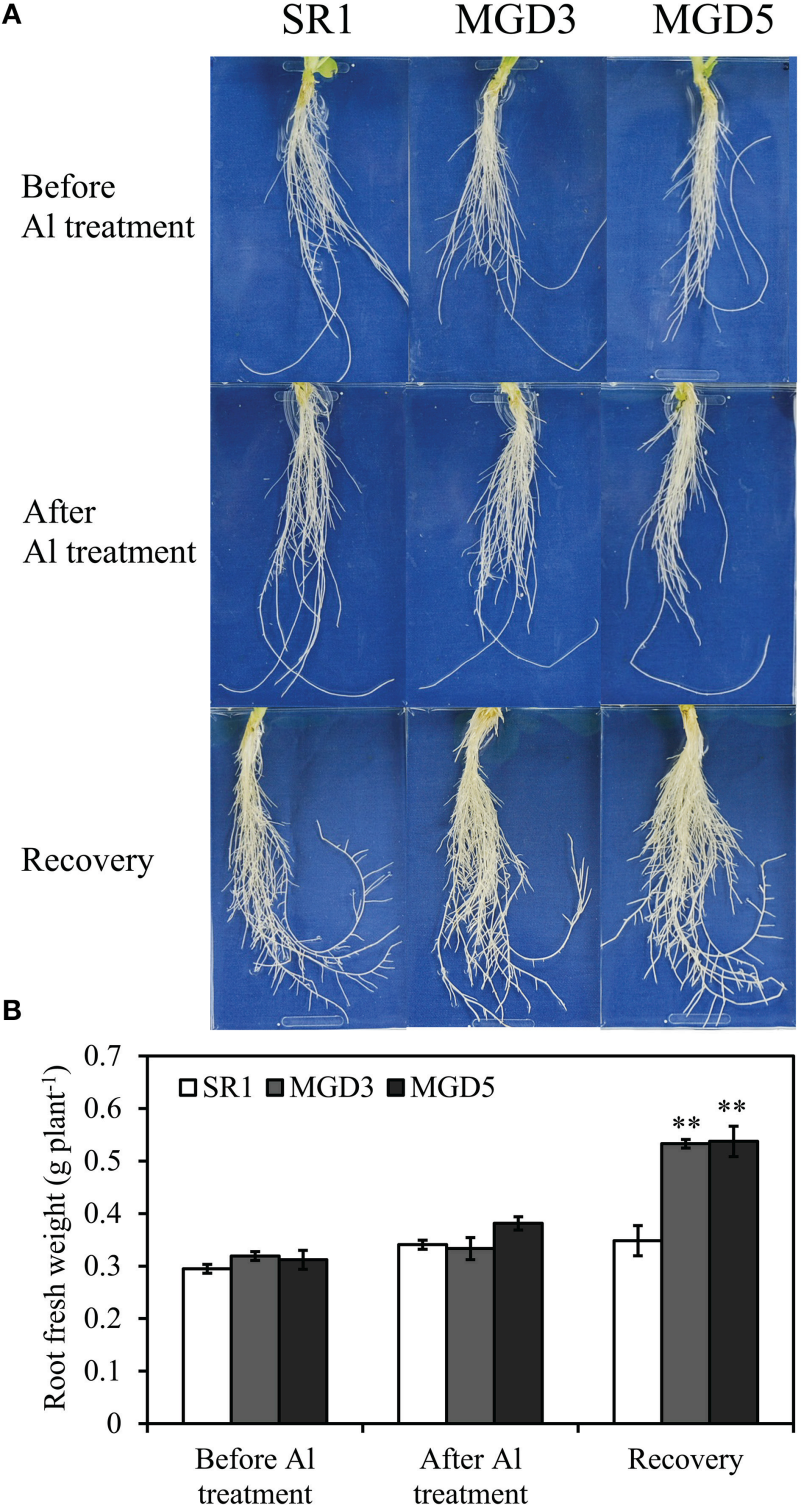
**Effect of Al treatment on root growth of wild-type SR1 and transgenic lines MGD3 and MGD5**. Tobacco seedlings at five- to six-leaf stage grown hydroponically on one-sixth-strength Hoagland solution was treated with 0 or 500 μM AlCl_3_ for 24 h. Photos were taken at each time point of tobacco roots **(A)** before and after Al treatment, 3 days after removal of AlCl_3_ for recovery. For fresh weight determination **(B)**, roots were collected from the plants either before or after Al treatment, after the 3 days' recovery. The experiment was repeated three times and each treatment included three replications. Data are means ± SE (*n* = 3). Asterisk indicates a significant difference (LSD test, ^**^P < 0.01) between wild-type and transgenic plants.

To examine whether *OsMGD* overexpression improves Al tolerance in tobacco plants, root growth was compared between wild-type SR1 and the transgenic lines MGD3 and MGD5 before and after Al treatment and 3 days after the removal of AlCl_3_. There was no difference in root growth between the wild-type and transgenic plants before Al treatment or after 24 h of Al treatment. Three days after the removal of AlCl_3_, however, root growth of MGD3 and MGD5 recovered quickly, the root fresh weights increased by 60.2% and 40.9%, respectively, but no such increase in root growth was seen in wild-type SR1 (Figures 1A,B). The root fresh weights in MGD3 and MGD5 were 53.0% (*P* < 0.01) and 54.3% (*P* < 0.01) higher than that of wild-type SR1.

### Al accumulation in root tips

**Figure 2:**
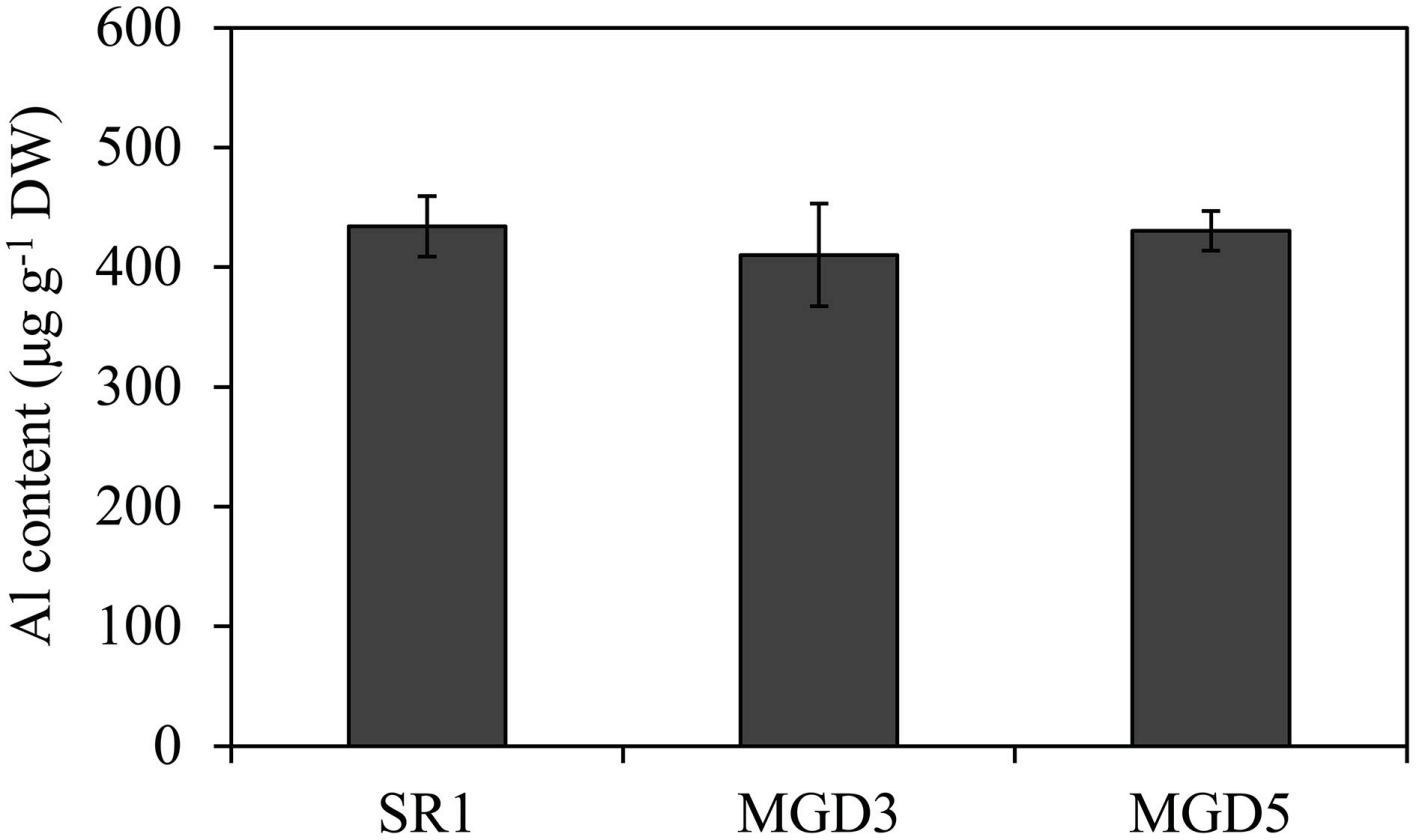
**Al content in root tips of wild-type SR1 and transgenic lines MGD3 and MGD5**. After Al treatment, root tips of plants exposed to 500 μM AlCl_3_ for 24 h were washed and dried to measure Al content by ICP-AES. The experiment was repeated three times and each treatment included three replications. Data are means ± SE (*n* = 3).

After exposed to 500 μM AlCl_3_ for 24 h, no difference in Al content was observed in the root tips of SR1 and the transgenic lines MGD3 and MGD5 (Figure 2), indicating that overexpression of *OsMGD* has no effect on Al accumulation in roots.

### Plasma membrane integrity and lipid peroxidation in the roots

**Figure 3:**
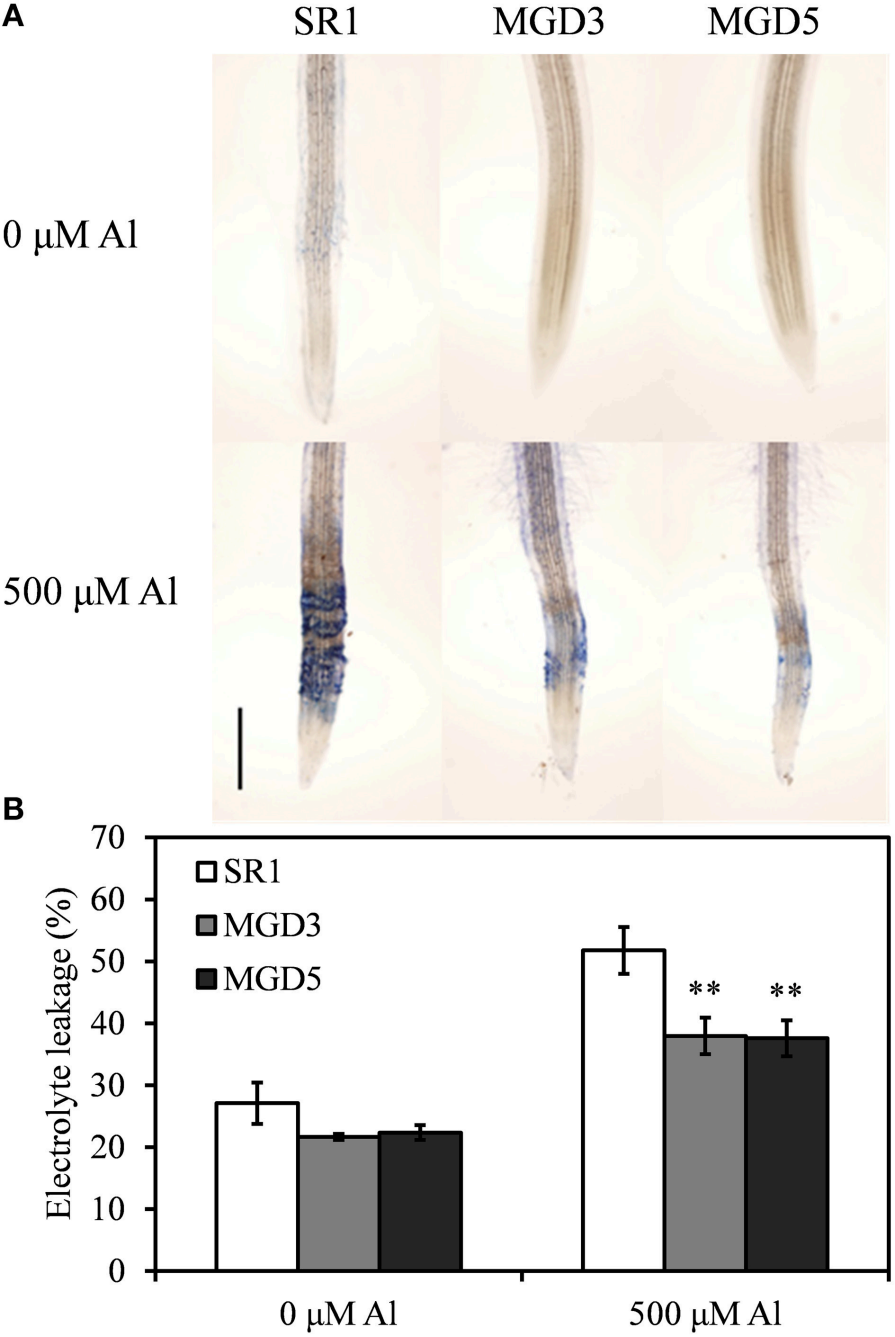
**Effect of Al treatment on plasma membrane integrity in the roots of wild-type SR1 and transgenic lines MGD3 and MGD5**. The root tips (0–20 mm) were stained with Evans blue **(A)** after exposed to 0 or 500 μM AlCl_3_ for 24 h, *Bar* indicates 500 μm. **(B)** Electrolyte leakage. The experiment was repeated three times and each treatment included three replications. Data are means ± SE (*n* = 3). Asterisk indicates a significant difference (LSD test, ^**^P < 0.01) between wild-type and transgenic plants.

The loss of plasma membrane integrity induced by Al was detected by Evans blue staining and electrolyte leakage assay (Figure 3). Root tips stained with Evans blue showed that there was quite small area and light Evans blue staining in plants without Al exposure, indicating the slight damage to the plasma membrane in plants without Al exposure. In Al-treated plants, however, the root tips of SR1 plants showed a more intense Evans blue staining than those of transgenic plants did, indicating serious damage to the plasma membrane integrity in wild-type tobacco plants (Figure 3A). To confirm the severity of membrane injury due to Al treatment, we measured electrolyte leakage in the root tips (0–20 mm from tip) (Figure 3B). Electrolyte leakage was strongly increased in SR1 (91.0%) and more weakly increased in MGD3 (75.3%) and MGD5 (68.1%). Thus, electrolyte leakage in the root tips of MGD3 and MGD5 were 26.7% (*P* < 0.01) and 27.4% (*P* < 0.01) lower than that of SR1, which indicating that the loss of membrane integrity under Al stress was alleviated in transgenic tobacco plants.

**Figure 4:**
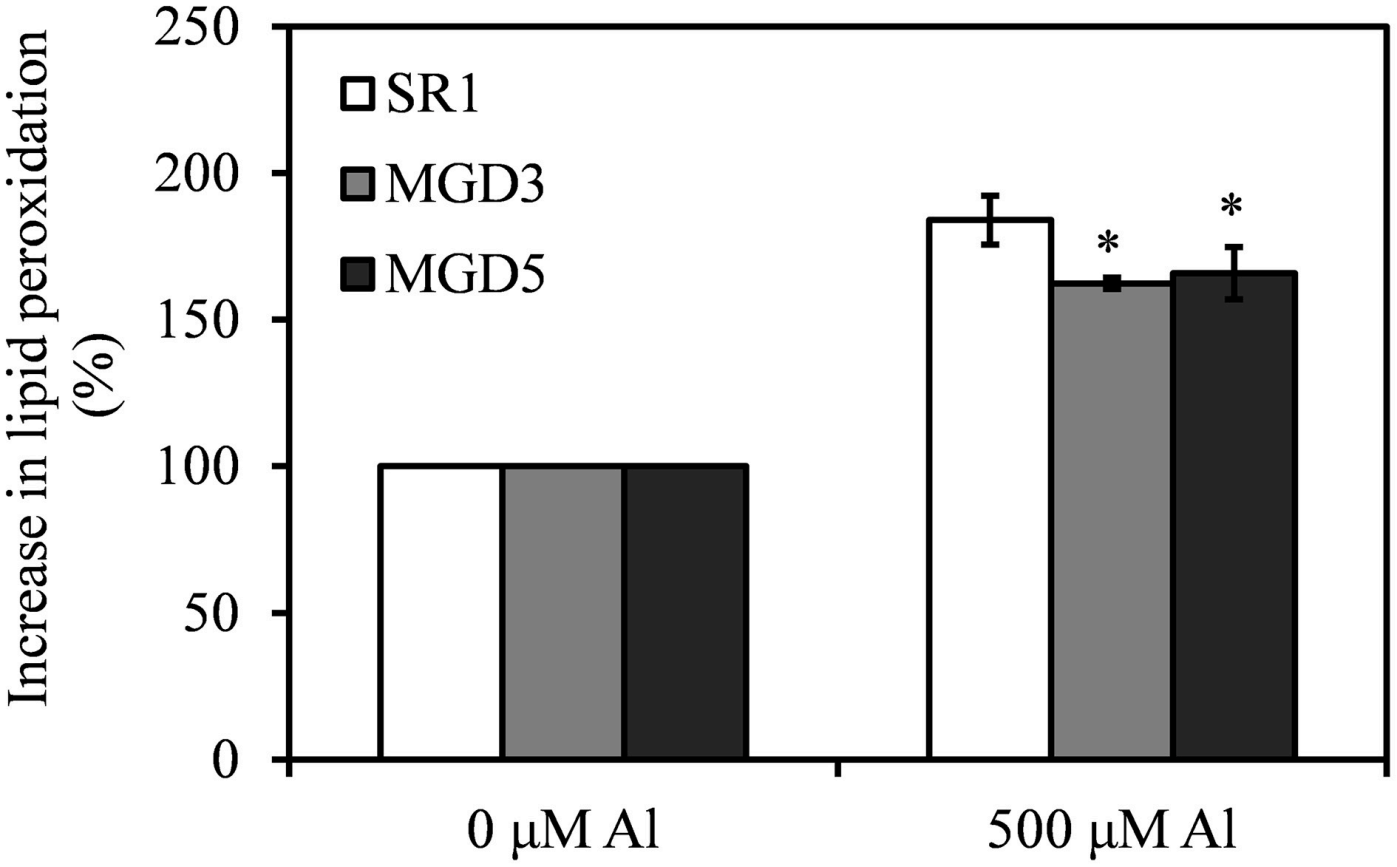
**Effect of Al treatment on the level of lipid peroxidation in the roots of wild-type SR1 and transgenic lines MGD3 and MGD5**. The level of lipid peroxidation was described as % increase in malondialdehyde (MDA), which was measured by the TBAR methods. The experiment was repeated three times and each treatment included three replications. Data are means ± SE (*n* = 3). Asterisk indicates a significant difference between wild-type and transgenic plants (LSD test, ^*^P < 0.05).

The level of MDA was used to assess lipid peroxidation in the plants. Under Al stress, MDA contents were increased in roots of all tested plants, but transgenic plants accumulated less MDA than wild-type plants did (Figure 4).

### Distribution of lipid classes

**Figure 5:**
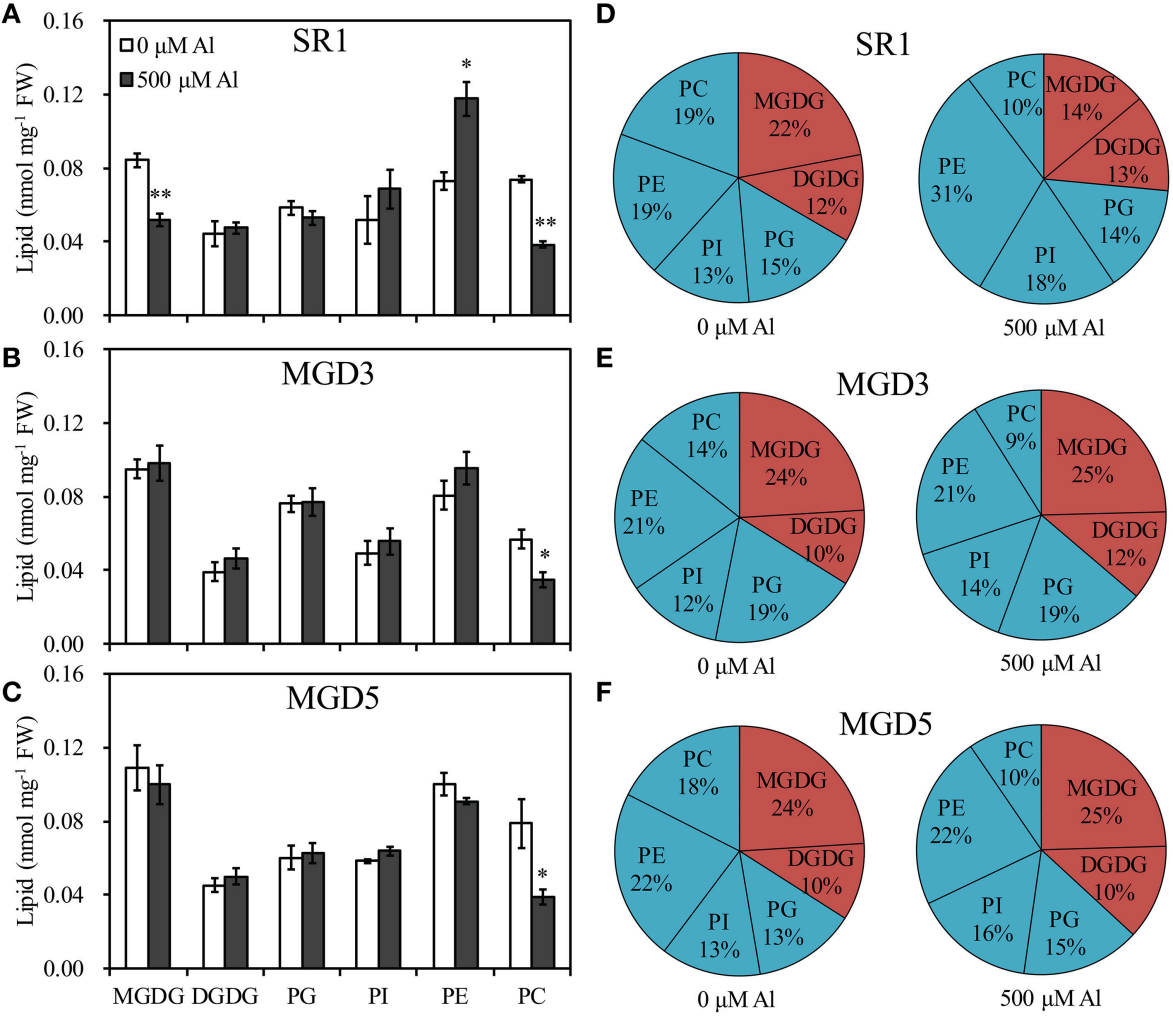
**Effect of Al treatment on the contents and the proportion of lipid in the roots of wild-type SR1 (A,D) and transgenic lines MGD3 (B,E) and MGD5 (C,F)**. Root tips (0–30 mm) were sampled from plants exposed to 0 or 500 μM AlCl_3_ for 24 h. Lipid was separated using TLC and quantified by gas chromatography. MGDG, monogalactosyldiacylglycerol; DGDG, digalactosyldiacylglycerol; PG, phosphatidylglycerol; PI, phosphatidylinositol; PE, phosphatidylethanolamine; PC, phosphatidylcholine; FW, fresh weight. Data are means ± SE (*n* = 3). Asterisk indicates a significant difference between treated and control plants (LSD test, ^*^P < 0.05, ^**^P < 0.01).

**Figure 6:**
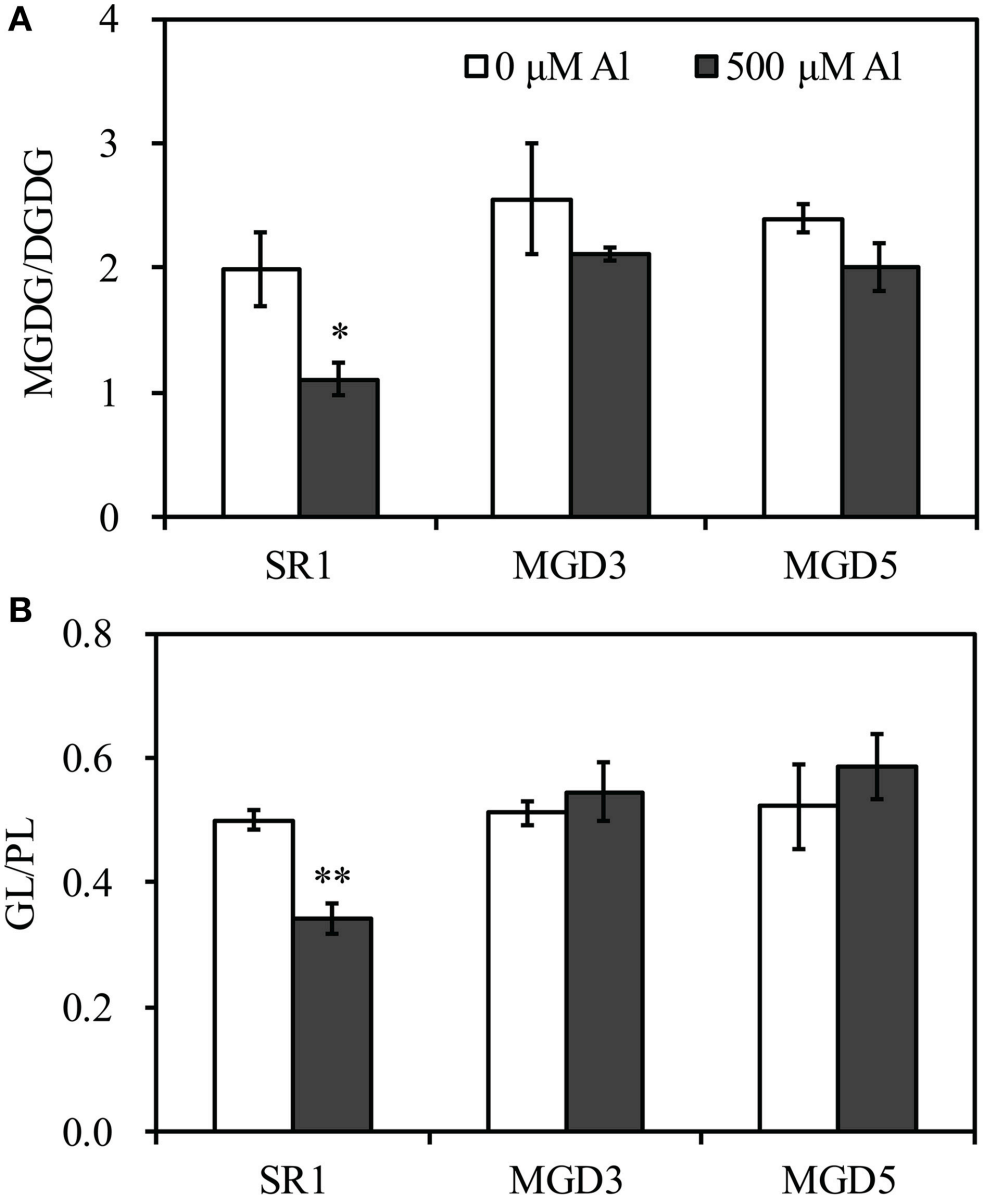
**Effect of Al treatment on the ratio of MGDG to DGDG (A) and the ratio of galactolipids to phospholipids (B) in the roots of wild-type SR1 and transgenic lines MGD3 and MGD5**. Root tips (0–30 mm) were sampled from plants exposed to 0 or 500 μM AlCl_3_ for 24 h. MGDG, monogalactosyldiacylglycerol; DGDG, digalactosyldiacylglycerol, GL, galactolipids; PL, phospholipids. Data are means ± SE (*n* = 3). Asterisk indicates a significant difference between treated and control plants (LSD test, ^*^P < 0.05, ^**^P < 0.01).

To elucidate the influence of *OsMGD* on Al tolerance, the lipid compositions of wild-type SR1 and transgenic plants were determined. Under control condition, MGDG contents showed slightly higher in transgenic plants than that in SR1 (Figure 5). After Al treatment, a significant decrease in the amount of MGDG in roots of SR1 was observed, which declined by 38.5% in the roots (Figure 5A). In the transgenic plants, however, the amount of MGDG remained stable (Figures 5B,C). The MGDG contents in MGD3 and MGD5 were 89.1% (*P* < 0.01) and 92.8% (*P* < 0.01) higher than that of SR1 under Al stress. In contrast, the DGDG showed no significant change under Al stress in all tested plants. Al stress also affected phospholipid compositions. PE was largely increased by 61.4% in wild-type SR1 after Al treatment. In contrast, no significant difference was observed in PE contents in transgenic plants. In all plants, Al caused decrease in PC contents, by 47.9% in SR1, 38.4% in MGD3 and 50.5% in MGD5. PG and PI did not change with Al exposure. Similar results were also found in leaves (Supplementary Figure 2). Furthermore, the proportion of total galactolipids (MGDG and DGDG) decreased while that of total phospholipids (PG, PE, PI, and PC) increased significantly in wild-type plants (Figure 5D). In transgenic plants, however, the proportions of galactolipids and phospholipids showed no significant changes in response to Al treatment (Figures 5E,F). Similar effects of Al on lipid proportion were also found in leaves (Supplementary Figures 2D–F). Also, the effect of Al stress on changes in fatty acid composition (mol %) was similar between wild-type SR1 and transgenic plants in both leaves and roots (Table 1, Supplementary Table 1). In addition, we did not measure the contents of phosphatidylserine, phosphatidic acid and lysophospholipids in this study, because they account for a small proportion of lipids (less than 10%, Zhao et al., 2011) and can hardly be separated and quantified by TLC and GC. For the ratio of MGDG/DGDG, it was significantly decreased under Al treatment in wild-type SR1 by 44.5%; while in transgenic plants, it showed no change (Figure 6A). The MGDG/DGDG ratio in MGD3 and MGD5 were 91.1% (*P* < 0.01) and 81.4% (*P* < 0.01) higher than that of SR1. Similar results were also found in leaves (Supplementary Figure 3A).

**Table 1:** **Effect of Al treatment on the fatty acid composition (mol%) in the lipid classes of the roots of wild-type SR1 and transgenic lines MGD3 and MGD5**.

**Lipid**	**Lines**	**Al (μM)**	**C16:1**	**C18:1**	**C18:2**	**C18:3**
MGDG	SR1	0	12.9±3.3	4.7±0.9	18.6±1.1	61.4±2.7
		500	2.6±0.3	11.4±1.8	15.1±0.8	66.9±2.3
	MGD3	0	8.0±1.5	6.0±1.9	20.0±2.8	63.7±2.4
		500	6.6±2.0	17.7±2.9	16.3±2.7	58.7±8.2
	MGD5	0	15.3±2.2	9.2±2.4	18.6±2.2	55.5±2.9
		500	7.7±2.2	11.8±1.4	11.7±3.1	70.0±2.3
DGDG	SR1	0	15.0±2.2	9.8±2.5	16.6±0.8	42.6±0.5
		500	6.8±1.8	13.1±2.4	12.6±1.4	49.2±4.9
	MGD3	0	18.9±6.7	7.0±2.8	19.6±3.3	36.8±4.8
		500	10.1±1.6	14.4±1.9	11.8±3.3	46.9±2.3
	MGD5	0	14.8±2.5	10.6±2.6	18.5±3.3	40.2±7.3
		500	8.4±1.0	15.9±1.1	13.7±1.9	44.0±3.5
PG	SR1	0	6.1±0.9	6.7±0.4	42.9±6.8	20.5±4.5
		500	7.3±2.0	9.6±2.6	48.9±4.8	12.0±1.1
	MGD3	0	16.2±2.7	8.5±1.1	39.7±2.2	13.9±0.9
		500	22.7±2.4	19.7±0.6	29.4±2.1	7.4±0.6
	MGD5	0	10.4±3.2	8.1±1.4	46.0±3.7	14.8±1.3
		500	28.5±1.9	9.2±2.1	33.5±1.6	7.8±1.5
PI	SR1	0	8.3±1.3	7.9±1.5	49.9±2.9	12.7±0.9
		500	18.4±2.7	8.3±0.8	45.0±4.8	6.1±0.6
	MGD3	0	17.5±2.8	16.8±3.4	31.1±5.4	12.9±2.5
		500	8.9±1.8	9.9±1.1	53.4±1.0	7.0±1.2
	MGD5	0	19.1±3.8	8.3±2.9	46.1±4.4	9.1±1.7
		500	9.8±1.1	14.2±1.9	48.1±0.2	6.9±0.8
PE	SR1	0	14.9±0.5	11.0±4.1	23.8±3.1	8.9±0.3
		500	6.1±2.3	15.0±0.2	25.5±1.8	8.5±0.4
	MGD3	0	6.6±1.4	5.5±1.3	26.2±3.0	5.8±0.3
		500	5.6±3.1	10.9±4.0	29.8±1.4	10.7±0.9
	MGD5	0	14.9±3.8	8.1±2.1	28.9±0.3	5.8±0.2
		500	4.1±0.7	15.9±0.8	29.7±1.0	9.2±0.2
PC	SR1	0	20.8±5.3	9.5±0.4	33.3±4.0	11.2±1.3
		500	5.1±1.2	5.5±0.5	41.2±2.3	10.9±0.5
	MGD3	0	17.1±3.6	10.1±2.3	34.9±2.9	12.3±0.7
		500	12.0±2.3	14.7±3.2	41.4±3.9	7.2±1.7
	MGD5	0	16.2±2.4	13.6±4.1	34.1±2.8	11.4±1.6
		500	18.±4.7	15.7±2.9	34.7±5.2	6.4±0.7

*Data are means ± SE (n = 3)*.

It has been proposed that the replacement of phospholipids, which are the major source of negative charge in the membrane system, with electrically neutral membrane lipids is one of the important strategies by which plants improve their Al tolerance (Wagatsuma et al., 2015). To assess this possibility, the ratio of electrically neutral galactolipids (GL) to anionic phospholipids (PL) in wild-type and transgenic plants was calculated (Figure 6B). In the roots, a significant decrease (31.8%) in the GL/PL ratio was observed in SR1 in response to Al treatment while no significant change was found in transgenic plants. The GL/PL ratio in MGD3 and MGD5 were 59.8% (*P* < 0.01) and 71.8% (*P* < 0.01) higher than that of SR1. Both in leaves and in roots, the GL/PL ratio after Al exposure was higher in transgenic plants than in SR1 (Figure 6B and Supplementary Figure 3B).

## Discussion

Al toxicity is a major factor limiting crop production in acidic soils. Inhibition of root growth is the primary symptom of Al toxicity which has been widely accepted as a suitable indicator for assessing Al tolerance in plants (Delhaize and Ryan, 1995; Ezaki et al., 2000; Tahara et al., 2008; Yin et al., 2010a,b). In the present study, transgenic plants overexpressing *OsMGD* showed rapid recovery of root growth after the removal of Al compared with the wild type (Figure 1), indicating that Al tolerance was enhanced in transgenic plants. Moreover, transgenic plants showed less damage of the membrane integrity and lower lipid peroxidation than the wild type (Figures 3, 4), which demonstrated that the Al tolerance in transgenic plants was enhanced. Additionally, no significant difference was found in Al^3+^ accumulation in root tips between transgenic and wild-type plants (Figure 2). These results suggest that maintenance of membrane integrity may contribute to the enhanced Al tolerance in transgenic plants.

Previous studies have shown that the primary mechanism by which Al affects plant function is through perturbing the membrane properties, which largely depend on membrane lipid compositions (Wagatsuma et al., 2005; Ahn and Matsumoto, 2006; Choudhury and Sharma, 2014). In this study, different changes in lipid compositions and fatty acid compositions under Al stress were observed in wild-type and transgenic plants (Figures 5A–C and Table 1). In the roots of wild-type plants, the contents of MGDG decreased markedly, and the contents of PE increased, while no change was found in transgenic plants. The constant level of MGDG in transgenic plants indicated that overexpression of *OsMGD* enables plants to rapidly replenish MGDG, which was decreased significantly by exposure to Al. However, in the wild type, the decreased MGDG was compensated by the phospholipids, mainly PE, which led to an increase in phospholipids proportion. It has been reported that phospholipids, which are negatively charged within the membrane, have great affinity for the positively charged Al^3+^ (Khan et al., 2005; Kochian et al., 2005; Wagatsuma et al., 2005; Huynh et al., 2012). The binding of Al^3+^ to phospholipids could lead to membrane rigidification, lateral lipid phase separation, and vesicle aggregation and fusion, finally lead to the decrease in membrane fluidity and increase in membrane permeability (Deleers et al., 1985, 1986). Moreover, it has been reported that the physical changes that result from the binding of Al^3+^ to phospholipids could also stimulate iron-induced lipid peroxidation (Oteiza, 1994), which could seriously damage membrane stability and integrity. In addition, it was found that the MGDG content was increased in Al tolerant wheat cultivar (Zhang et al., 1997), indicating that the MGDG could contribute to plant Al tolerance. These results showed that regulation of the level of MGDG is beneficial in maintaining the fundamental properties of the membrane under Al stress, and thus leads to improved Al tolerance. Similar results were found in rice that the lipid compositions were not changed in Al-resistant cultivars, while a sharp decrease in membrane lipid content was exhibited in Al-sensitive cultivars (Huynh et al., 2012). Substantial alterations in membrane lipid composition may also lead to conformational changes in membrane proteins, which play vital roles in plant metabolic activities (Navari-Izzo et al., 2000). Therefore, stability of membrane lipids may help transgenic plants maintain proper physiological functions of its membrane system, and thus contribute to rapid regrowth of the roots after removal of Al stress.

In addition, it was noticed that under normal condition, the contents of MGDG were slightly higher in transgenic plants than the wild type, but the proportion of MGDG showed no difference between transgenic and wild-type plants (Figure 5). This was because the MGDG content was low in roots, the slightly increase of MGDG can hardly affect its proportion of the total lipid. And more important, the total lipid content was increased in the transgenic plants, comparing with the wild type under normal condition, so the proportion of MGDG showed no difference between transgenic and wild-type plants.

Regulating the ratio of MGDG to DGDG is another strategy by which the plant can maintain the biochemical and physiological properties of its membranes under stress conditions (Chaffai et al., 2007; Gaude et al., 2007; Torres-Franklin et al., 2007). In the present study, a significant decrease in the MGDG/DGDG ratio was found in the wild type, while this ratio remained unchanged in transgenic plants under Al stress (Figure 6A). Similar changes in the MGDG/DGDG ratio were also found in wheat, where it showed that the MGDG/DGDG ratio was unaffected in an Al-resistant cultivar but significantly decreased in an Al-sensitive cultivar (Zhang et al., 1997). Likewise, the MGDG/DGDG ratio was unchanged in drought-tolerant species, though it decreased significantly in drought-sensitive species under drought stress (Olsson et al., 1996). A correlation between higher MGDG/DGDG ratio and salt resistance has also been reported in soybean (Zenoff et al., 1994). It was reported that MGDG was more susceptible to be degraded by galactolipases than other lipids (Skórzyńska et al., 1991), and this may be the reason for the reduction of the MGDG/DGDG ratio under stress conditions. Moreover, high DGDG levels could increase membrane permeability. It has been shown that membrane vesicles containing DGDG are more permeable to Rb^+^ than vesicles composed of PC are (Webb and Green, 1989). Therefore, maintaining DGDG at a low level could play an important role in limiting the uptake of toxic Al species and prevent Al-induced increases in membrane leakiness (Sasaki et al., 1994). Thus, the higher ratio of MGDG/DGDG seems to contribute to maintaining the membrane stability and integrity, leading to enhanced Al tolerance in transgenic plants.

In addition, it has been shown that the proportion of negative phospholipids tends to decrease under Al stress while that of the electrically neutral lipids tends to increase, which is a common strategy for protecting the membrane from Al toxicity in plants (Wagatsuma et al., 2015). Maejima et al. (2014) reported that a decrease in phospholipid contents and an increase in galactolipid contents lead to an enhanced Al tolerance in rice. In the present study, the ratio of galactolipids to phospholipids was calculated and a significantly decrease of this ratio was observed in wild-type plants, while no change was found in transgenic plants after exposure to Al stress (Figures 5D–F, 6B). The higher ratio of galactolipids to phospholipids in transgenic plants could facilitate better maintenance of membrane properties, and leads to enhanced Al tolerance.

In the present study, similar alteration in lipid composition was also observed in leaves under Al stress, which showed higher galactolipids contents, MGDG/DGDG and GL/PL ratios in transgenic plants than that in wild-type SR1 (Supplementary Figures 2, 3). As Al stress could also cause lipid transporting or remodeling in the whole plant (Chaffai et al., 2005; Huynh et al., 2012), the high contents of galactolipids in leaves may facilitate the increase in Al tolerance of the whole plant. Moreover, since there is significantly difference between wild-type and *OsMGD* transgenic plants in their Al tolerance, it is interesting and important to investigate the expression of *OsMGD* gene under Al stress treatment in the plant of rice, to further clarify the regulation of MGDG biosynthesis under Al stress.

In conclusion, overexpression of *OsMGD* in tobacco could help plants to maintain the biochemical and physiological properties of membranes under Al stress by keeping membrane lipid compositions and MGDG/DGDG ratio constant, thereby enabling better root growth under the condition of Al stress. Our results also indicate that the regulation of galactolipid biosynthesis by overexpression of *OsMGD* plays an important role in maintaining membrane integrity under Al stress, which provides us with a new strategy for improving Al tolerance in plants. In addition, changing the activity of galactolipase could also affect the MGDG level. It has been reported that under Cd stress, the activity of galactolipase was increased which was accompanied with the decrease of MGDG level (Skórzyńska et al., 1991). Therefore, future studies that alter the expression of galactolipase should be conducted to clarify whether MGDG and galactolipase activation are the key to Al damage and sensitivity.

## Author contributions

XD and LY planned experiment. MZ and LY conducted experiment, collected and analyzed the data, and prepared the draft. LQ and XW helped measurements of membrane lipids. SW and HL helped in drafting the manuscript and interpretation the results.

## Funding

This work was supported by the West Light Foundation of the Chinese Academy of Sciences, the Youth Innovation Promotion Association of the Chinese Academy of Sciences (No. 2015389), and the National Natural Sciences Foundation of China (No. 31200206).

### Conflict of interest statement

The authors declare that the research was conducted in the absence of any commercial or financial relationships that could be construed as a potential conflict of interest.
