# Supplementary material for: Regulation of Galactolipid Biosynthesis by Overexpression of the Rice MGD Gene Contributes to Enhanced Aluminum Tolerance in Tobacco
Source: Front Plant Sci. 2016 Mar 30;7:337. doi: 10.3389/fpls.2016.00337 (PMC4811928; doi:10.3389/fpls.2016.00337)
Supplement: Supplementary file 1 [file Image1.PDF]

Supplementary Material:

Regulation of galactolipid biosynthesis by overexpression of the rice *MGD* gene contributes to enhanced aluminum tolerance in tobacco

Meijuan Zhang[1,2], Xiping Deng[1,2,3*], Lina Yin[2,3,4*], Lingyun Qi[4], Xinyue Wang[3], Shiwen Wang[2,3], Hongbing Li[2,3]

* Corresponding author: Xiping Deng and Lina Yin

Dr. Lina Yin

State Key Laboratory of Soil Erosion and Dryland Farming on the Loess Plateau, Institute of Soil and Water Conservation, Northwest A&F University, Xinong Road No. 26, Yangling, Shaanxi, China. 712100.

Phone: +86-29-87012872; Fax: +86-29- 87012210

E-mail address: linayin@nwsuaf.edu.cn

Supplementary Figures

Supplementary Figure 1. Lipid composition in tobacco leaves separated by TLC.

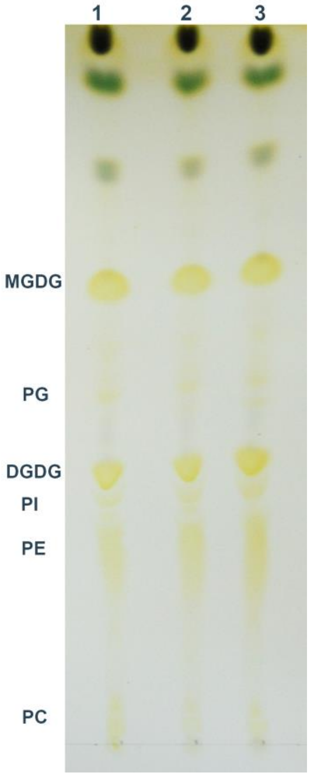

Supplementary Figure 1 | Lipid composition in tobacco leaves. The lipids were extracted from tobacco leaves and separated by TLC, and stained with iodine. MGDG, monogalactosyldiacylglycerol; PG, phosphatidylglycerol; DGDG, digalactosyldiacylglycerol; PI, phosphatidylinositol; PE, phosphatidylethanolamine; PC, phosphatidylcholine.

Supplementary Figure 2 | Effect of Al treatment on the contents of lipid composition and the proportion of lipid classes in the leaves.

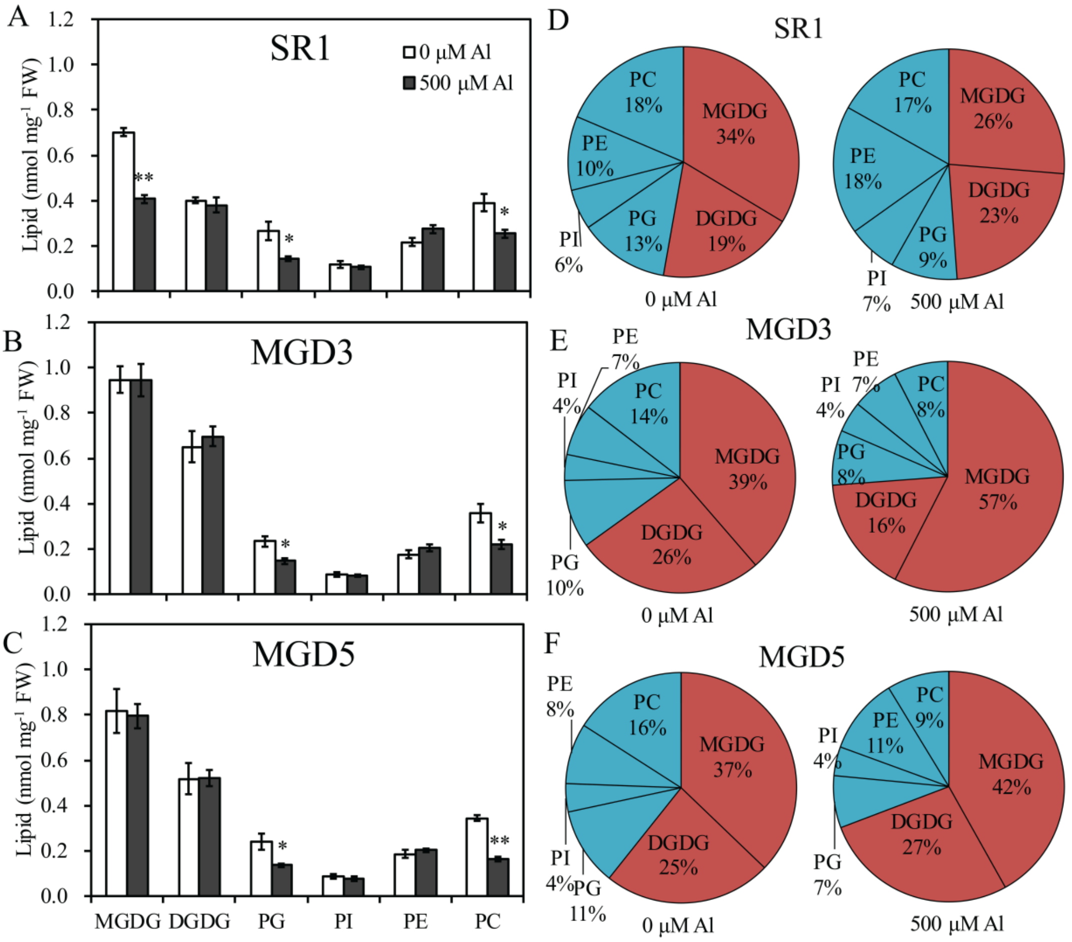

Supplementary Figure 2 | Effect of Al treatment on the contents and the proportion of lipid in the leaves of wild-type SR1 (A, D) and transgenic lines MGD3 (B, E) and MGD5 (C, F). Leaves were sampled from plants exposed to 0 or 500 μM $AlCl_3$ for 24 hours. MGDG, monogalactosyldiacylglycerol; DGDG, digalactosyldiacylglycerol; PG, phosphatidylglycerol; PI, phosphatidylinositol; PE, phosphatidylethanolamine; PC, phosphatidylcholine; FW, fresh weight. Data are means ± SE (n = 3). Asterisk indicates a significant difference between treated and control plants (LSD test, $^{*}P < 0.05$, $^{**}P < 0.01$).

Supplementary Figure 3. Effect of Al treatment on the ratio of MGDG to DGDG (A) and the ratio of galactolipids to phospholipids (B) in the leaves of wild-type SR1 and transgenic lines MGD3 and MGD5.

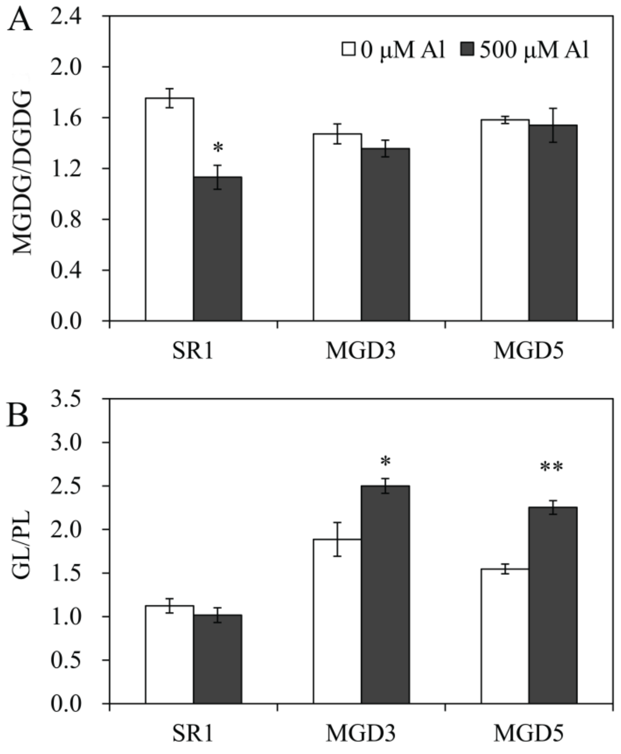

Supplementary Figure 3 | Effect of Al treatment on the ratio of MGDG to DGDG (A) and the ratio of galactolipids to phospholipids (B) in the leaves of wild-type SR1 and transgenic lines MGD3 and MGD5. Leaves were sampled from plants exposed to 0 or 500 µM $AlCl_3$ for 24 hours. MGDG, monogalactosyldiacylglycerol; DGDG, digalactosyldiacylglycerol, GL, galactolipids; PL, phospholipids. Data are means ± SE (n = 3). Asterisk indicates a significant difference between treated and control plants (LSD test, $^{*}P < 0.05$, $^{**}P < 0.01$).

Supplementary Table 1 Effect of Al treatment on the fatty acid composition (mol%) in the lipid classes of the leaves of wild-type SR1 and transgenic lines MGD3 and MGD5.

Lipid	Lines	Al (μM)	C16:1	C18:1	C18:2	C18:3
MGDG	SR1	0	13.2 ± 2.8	20.2 ± 3.9	13.2 ± 1.2	51.3 ± 4.9
		500	23.6 ± 2.2	18.5 ± 3.5	19.5 ± 2.3	37.3 ± 4.1
	MGD3	0	18.8 ± 1.8	10.0 ± 0.7	23.0 ± 1.0	44.1 ± 1.0
		500	24.0 ± 1.2	26.3 ± 3.3	17.3 ± 1.8	28.9 ± 2.2
	MGD5	0	19.7 ± 1.7	17.6 ± 1.0	21.9 ± 1.8	47.0 ± 2.1
		500	30.0 ± 1.3	10.4 ± 2.4	16.5 ± 1.8	40.4 ± 4.2
DGDG	SR1	0	23.0 ± 2.0	18.2 ± 3.9	10.9 ± 0.9	30.8 ± 4.3
		500	12.1 ± 2.1	18.4 ± 2.0	12.0 ± 0.7	41.2 ± 1.0
	MGD3	0	22.6 ± 1.4	11.6 ± 1.5	12.4 ± 1.0	34.3 ± 1.8
		500	20.6 ± 3.7	10.5 ± 0.9	11.8 ± 1.8	38.8 ± 2.0
	MGD5	0	27.0 ± 4.2	15.7 ± 2.3	13.7 ± 2.7	42.1 ± 1.4
		500	13.6 ± 1.0	11.0 ± 1.8	12.1 ± 3.1	45.4 ± 3.9
PG	SR1	0	6.7 ± 2.3	13.0 ± 0.8	12.6 ± 2.2	46.6 ± 3.0
		500	11.8 ± 1.8	16.0 ± 2.4	9.9 ± 1.5	42.2 ± 3.0
	MGD3	0	13.4 ± 1.7	12.2 ± 0.8	11.7 ± 1.9	39.6 ± 1.1
		500	11.1 ± 2.7	13.1 ± 2.3	8.9 ± 0.1	44.4 ± 2.5
	MGD5	0	14.9 ± 2.8	11.0 ± 0.5	11.3 ± 0.3	42.2 ± 3.1
		500	21.0 ± 2.0	22.1 ± 2.5	10.2 ± 1.8	24.0 ± 2.4
PI	SR1	0	17.3 ± 1.7	12.3 ± 1.9	24.2 ± 4.1	25.0 ± 0.8
		500	30.0 ± 2.7	7.4 ± 1.1	7.3 ± 0.8	35.0 ± 4.4
	MGD3	0	15.2 ± 3.6	12.8 ± 2.5	24.0 ± 2.5	24.8 ± 3.2
		500	20.4 ± 3.9	7.7 ± 1.0	9.7 ± 1.2	39.8 ± 4.0
	MGD5	0	18.4 ± 0.5	18.5 ± 3.3	24.9 ± 2.1	17.5 ± 2.6
		500	25.2 ± 1.7	10.3 ± 0.5	10.2 ± 1.0	32.5 ± 1.3
PE	SR1	0	9.7 ± 1.8	12.4 ± 0.5	10.4 ± 1.5	26.2 ± 2.3
		500	16.4 ± 1.9	11.6 ± 1.5	6.6 ± 0.3	24.8 ± 1.1
	MGD3	0	21.7 ± 3.2	9.0 ± 2.4	6.3 ± 1.2	20.0 ± 0.5
		500	22.6 ± 3.9	7.7 ± 0.8	12.5 ± 3.1	14.9 ± 1.5
	MGD5	0	11.3 ± 1.3	13.3 ± 2.2	12.2 ± 3.2	22.4 ± 1.5
		500	17.9 ± 0.8	17.5 ± 2.7	10.9 ± 1.9	12.0 ± 1.2
PC	SR1	0	9.7 ± 0.9	12.4 ± 0.2	10.4 ± 1.3	26.2 ± 1.0
		500	16.4 ± 3.8	11.6 ± 2.6	6.6 ± 1.5	24.8 ± 2.2
	MGD3	0	15.0 ± 2.4	13.2 ± 0.8	21.3 ± 1.8	23.5 ± 1.5
		500	28.2 ± 1.8	12.8 ± 0.3	15.0 ± 1.3	17.6 ± 1.3
	MGD5	0	13.1 ± 1.9	15.6 ± 2.0	24.4 ± 2.6	22.3 ± 1.1
		500	17.1 ± 2.1	22.9 ± 2.5	15.4 ± 0.6	18.8 ± 0.8

Data are means ± SE (n = 3).
